# Supplementary material for: MetaRibo-Seq measures translation in microbiomes
Source: Nat Commun. 2020 Jun 29;11:3268. doi: 10.1038/s41467-020-17081-z (PMC7324362; doi:10.1038/s41467-020-17081-z)
Supplement: Supplementary file 10 — Supplementary Data 7 [file 41467_2020_17081_MOESM10_ESM.zip › File2/Confidence_High_Taxonomy/176797_out.krona.html]

Javascript must be enabled to view this page.

members
magnitude
magnitudeUnassigned
count
unassigned
taxon
rank

176797\_out

2

2
2
superkingdom

phylum
2
1239

species
1

SRS042966\_contig\_number\_17504
1262992

909932
1
class

order
1
1843489

family
1
31977

genus
1
29465

1

SRS893350\_contig\_number\_13299
species
2292078
